# Activation of cGMP/PKG/p65 signaling associated with PDE5‐Is downregulates CCL5 secretion by CD8 ^+^ T cells in benign prostatic hyperplasia

**DOI:** 10.1002/pros.23801

**Published:** 2019-04-08

**Authors:** Song Jin, Peng Xiang, Jie Liu, Yang Yang, Shuai Hu, Jindong Sheng, Qun He, Wei Yu, Wenke Han, Jie Jin, Jing Peng

**Affiliations:** ^1^ Department of Urology Peking University First Hospital and Institute of Urology, Peking University Beijing China; ^2^ National Research Center for Genitourinary Oncology Beijing China; ^3^ Beijing Key Laboratory of Urogenital Diseases (male) Molecular Diagnosis and Treatment Center Beijing China

**Keywords:** benign epithelial cell, CCL5, CD8^+^ T cell, cyclic guanosine monophosphate/protein kinase G, nuclear factor‐κB/p65

## Abstract

**Background:**

Benign prostatic hyperplasia (BPH) is the most common urological disease in elderly men, but the underlying pathophysiological mechanisms are complex and not fully understood. Phosphodiesterase type 5 inhibitors (PDE5‐Is) used to treat BPH could upregulate the cyclic guanosine monophosphate (cGMP)‐dependent protein kinase G (PKG) signaling, which was shown to blunt inflammation in the prostate. Our previous findings indicate that CD8^+^ T cells promote the proliferation of BPH epithelial cells (BECs) in low androgen conditions through secretion of CCL5; however, the role of the cGMP/PKG pathway in the process is unclear.

**Methods:**

Paraffin‐embedded tissues were used for expression quantity of CD8^+^ T cells, CCL5, cyclin D1, and PDE5 protein by immunohistology in prostate specimens which were/were not treated with finasteride 5 mg daily for at least 6 months before surgery. BPH‐1 cells were cocultured with or without CD8 ^+^ T cells or PDE5‐Is in low androgen conditions for 4 days. The conditioned media, BPH‐1 cells, and CD8 ^+^ T cells were harvested for the subsequent experiments. The quantitative polymerase chain reaction was used for assaying the level of messenger RNA expression of CCL5. CCL5 in the conditioned media was detected by the enzyme‐linked immunosorbent assay. The effect of PDE5‐Is on cocultured BPH‐1/CD8 ^+^ T‐cell proliferation was detected by the cell counting kit‐8. A high‐fat diet (HFD)‐induced prostatic hyperplasia rat model was used to investigate the effect of cGMP/PKG activation in CD8 ^+^ T cells in vivo.

**Results:**

CD8^+^ T‐cell infiltration into human BPH tissues was positively correlated with the expression of CCL5, cyclin D1, and PDE5, whereas in an HFD‐induced prostatic hyperplasia rat model, the activation of the cGMP/PKG signaling by a PDE5‐I could suppress the CD8 ^+^ T‐cell infiltration and the CCL5 and cyclin D1 expression. Furthermore, the activation of the cGMP/PKG pathway inhibited CCL5 secretion by CD8 ^+^ T cells by downregulating nuclear factor‐κB p65 phosphorylation, which reduced the growth of BPH‐1 through CCL5/STAT5/CCND1 signaling.

**Conclusions:**

Our results indicate that the upregulation of the cGMP/PKG/p65 signaling reduces CCL5 secretion in CD8 ^+^ T cells, which in turn decreases the proliferation of BECs in low androgen conditions, suggesting that the combination of 5α reductase inhibitors lowering androgen levels and PDE5‐Is may be a novel, more effective treatment for BPH patients.

## 1 INTRODUCTION

Lower urinary tract symptoms (LUTS) associated with benign prostatic hyperplasia (BPH) are common in the aging male population and their prevalence is increasing; however, the pathophysiological mechanisms underlying BPH development are complex and poorly understood.^1^ Several clinical trials have demonstrated that phosphodiesterase type 5 inhibitors (PDE5‐Is) could reduce the International Prostate Symptom Score, storage and voiding LUTS, and improve quality of life.^2^, ^3^, ^4^, ^5^ Most studies suggested that the clinical effect of PDE5‐Is is associated with cyclic guanosine monophosphate (cGMP) signaling through cGMP‐dependent protein kinase G (PKG), which mainly reduces smooth muscle tone of the detrusor, prostate, and urethra, thus relieving LUTS.^6^, ^7^, ^8^

Dihydrotestosterone synthesized from testosterone by 5α‐reductase (5AR) II plays a critical role in prostate growth, and a 5AR inhibitor (5AR‐I) finasteride is known to alleviate BPH symptoms. In our previous studies, we have shown that BPH tissues from finasteride‐treated patients had increased CD8^+^ T‐cell infiltration,^9^ which could promote the proliferation of BPH epithelial cells (BECs) in low androgen conditions by secreting a chemokine CCL5 (also known as RANTES).^10^ Moreover, we also found that PDE5 protein expression positively correlated with CD8^+^ T‐cell infiltration and CCL5 and cyclin D1 levels in human BPH tissues, whereas activation of cGMP/PKG signaling could suppress CD8^+^ T‐cell infiltration and the expression of CCL5 and cyclin D1 in preliminary experiments using a high‐fat diet (HFD) BPH rat model. Other studies have also reported that the cGMP/PKG signaling pathway may regulate the inflammatory response implicated in several pathological conditions, including BPH,^11^, ^12^, ^13^ but the mechanism of cGMP/PKG signaling and its specific role in inflammation in the prostate is not well understood. In particular, activation of the cGMP/PKG pathway in CD8^+^ T cells and its effect on BEC proliferation is unclear, and there are few reports on the relationship between the cGMP/PKG pathway and CCL5 secretion. Therefore, the aim of the present study was to clarify the involvement of the cGMP/PKG pathway in the regulation of CCL5 expression in CD8^+^ T cells and its effect on BEC proliferation in low androgen conditions.

## 2 MATERIALS AND METHODS

### 2.1 Patients

Patients were selected using the electronic medical record system containing the data on 921 patients with BPH who underwent transurethral resection of the prostate between January 2007 and December 2011 in the Department of Urology, Peking University First Hospital, Beijing, China, and it was approved by the ethical committee of our institution. Patients who had urinary tract infection, prostatitis, earlier prostate‐related surgery, or the history of urethral catheterization were excluded from this study. Prostate tissues were obtained from 34 BPH patients treated or not with finasteride (5 mg daily) for at least 6 months before surgery and examined microscopically by two pathologists to confirm the diagnosis of BPH not associated with prostate cancer or prostatic intraepithelial neoplasia.

### 2.2 Animals

An HFD‐induced prostatic hyperplasia model was established as previously described.^14^, ^15^, ^16^ Male Sprague‐Dawley rats (7‐ to 9‐week old; 200‐220 g) (Beijing Keao Xieli Feed Co, Ltd, Beijing, China) were housed in individual cages under standard conditions in a temperature‐ and humidity‐controlled room with a 12‐hour light/dark cycle and had free access to food and water. After a week on a standard rat diet, animals were randomly assigned to control (*n* = 6), HFD (*n* = 6) or HFD + PDE5‐Is (*n* = 6) groups according to their weight. The control group continued on the regular diet (3.85 kcal/g; carbohydrate, 70%; protein, 20%; fat, 10%, kcal), HFD and HFD + PDE5‐Is groups were fed HFD (5.24 kcal/g; carbohydrate, 20%; protein, 20%; fat, 60%, kcal) (Beijing HFK Bioscience Co, Ltd, Beijing, China) for 16 weeks; furthermore, HFD + PDE5‐Is group (*n* = 6) was treated with tadalafil (2 mg/kg daily for 5 days a week by oral gavages) during the last 4 weeks. Blood samples were obtained from all animals via vena cava at week 12. Rats were killed by a lethal dose of pentobarbital (5% pentobarbital, 0.2 mL per 100 g by intraperitoneal injection) and prostate specimens were harvested and processed for subsequent analyses. Serum testosterone test was performed using an Automated Chemiluminescence System (Bayer Diagnostics, East Walpole, MA). All animal experiments were performed under the supervision and guidelines of the Peking University First Hospital Animal Care and Use Committee.

### 2.3 Reagents and antibodies

The following antibodies were used for immunohistochemistry (IHC) and Western blot analysis: rabbit anti‐CD8^+^ (ZA‐0508; ZSGB‐Bio, Beijing, China), rabbit anti‐CCL5 (ab9679), and anti‐PDE5 (ab14672) (both from Abcam, Cambridge, UK), mouse anti‐glyceraldehyde 3‐phosphate dehydrogenase (GAPDH) (60004‐1‐Ig) and anti‐β‐tubulin (66240‐1‐Ig) (both from Proteintech Group Inc, Chicago, IL), rabbit anti‐CCND1 (92G2), anti‐STAT5 (9363), anti‐phospho‐STAT5 (9314), anti‐NF‐κB p65 (8242), and anti‐phospho‐NF‐κB p65 (93H1) (all from Cell Signaling Technology, Denver, MA). Recombinant human (rh)CCL5 (278‐RN) and anti‐CCL5 neutralizing antibody (MAB678) were purchased from R&D systems (Minneapolis, MN), tadalafil (S1512) and a PKG inhibitor KT5823 (S4684) were obtained from Selleckchem (Houston, TX), and a PDE5‐insensitive PKG agonist Sp‐8‐Br‐PET‐cGMP was purchased from Biolog (Hayward, CA). All reagents were used in low androgen medium to reproduce the conditions of finasteride treatment.

### 2.4 Immunohistochemistry

Serial paraffin sections of the prostate samples were stained with specific antibodies using IHC as previously described.^10^ Quantitative analysis was performed by measuring integrated optical density using the Image‐Pro Plus 6.0 software (Media Cybernetics, Rockville, MD). The degree of prostatic CD8^+^ T‐cell infiltration was calculated as the mean number of CD8^+^ T‐cell aggregates in the prostate stroma observed under ×100 objective (CX31, Olympus Corporation, Tokyo, Japan). The expression intensity of CCL5, CCND1, and PDE5 protein was assessed semiquantitatively as integrated optical density under ×100 objective (Olympus Microscope). Each prostate sample was selected by five horizons. All analyses were performed by two experienced pathologists in a blinded fashion.

### 2.5 Cell lines and coculture experiments

BPH epithelial cell line BPH‐1 (KG1008; KeyGen Biotech Co, Ltd, Nanjing, China) and CD8^+^ T lymphocytic cell line Molt‐3 (CRL‐1552; American Type Culture Collection, Rockville, MD)^17^ were grown in Rosewell Park Memorial Institute (RPMI)‐1640 medium (SH30809.01B; HyClone, South Logan, UT) supplemented with 1% penicillin G, 1% streptomycin, and 10% fetal bovine serum (FBS) in a humidified incubator at the 5% CO_2_ atmosphere and 37°C. To create a low androgen environment, charcoal‐stripped FBS (SH30068.03; HyClone) and phenol red‐free medium were used.^10^ In coculture experiments, BPH‐1 cells (2 × 10^4^ cells per well) were seeded in low androgen medium with 10% charcoal‐treated FBS in the lower chamber of six‐well Transwell plates (3460/3450; Corning Inc, Corning, NY) with or without Molt‐3 cells (2 × 10^4^/well) seeded into the upper chamber containing polycarbonate membrane inserts (0.4‐μm pore size). Then, BPH‐1/Molt‐3 cell cocultures or BPH‐1 cell monocultures were incubated with or without tadalafil/Sp‐8‐Br‐PET‐cGMP and KT5823 for 4 days, and the conditioned medium, BPH‐1 cells, and Molt‐3 cells were harvested for analysis.

### 2.6 Cell proliferation assay

BPH‐1 cells were plated on 96‐well plates (800 cells per well) overnight. Then, the culture medium was changed for a 1:1 mixture of conditioned medium and fresh phenol red‐free medium with 10% charcoal‐stripped FBS supplemented or not with rhCCL5 or anti‐CCL5 neutralizing antibody and cells were incubated for 6 days. BPH‐1 cell growth was assessed at days 2, 4, and 6 using the Cell Counting Kit‐8 (CCK‐8) (CK04; Dojindo Molecular Technologies, Tokyo, Japan) according to the manufacturer's protocol.^10^

### 2.7 Western blot analysis

BPH‐1 cells were planted into the lower chamber of six‐well Transwell plates (5 × 10^3^ cells per well) and cultured in phenol red‐free RPMI‐1640 medium overnight. In the coculture group, Molt‐3 cells at 5 × 10^3^ cells per well were planted the next day into the upper chamber with 0.4‐μm pore polycarbonate membrane inserts. BPH‐1 and Molt‐3 cells were incubated for 3 days and collected for Western blot analysis performed as described previously.^10^

### 2.8 Quantitative polymerase chain reaction

Total RNA was extracted using TRIzol reagent (15596‐018; Invitrogen, Grand Island, NY). According to the manufacturer's protocol, complementary DNA (cDNA) was synthesized from 1 μg RNA, using a high‐capacity cDNA reverse transcription kit (4368813; Applied Biosystems, Foster, CA). Quantitative real‐time polymerase chain reaction (qRT‐PCR) was conducted using the GoTaq qPCR Master Mix (A6001; Promega, Madison, WI) for a two‐step cycling protocol with an Applied Biosystems 7500 Fast Real‐Time PCR system. The primers were CCL5, 5‘‐CCAGCAGTCGTCTTTGTCAC‐3′ (forward) and 5‘‐CTCTGGGTTGGCACACACTT‐3′ (reverse); GAPDH 5‘‐ACAACTTTGGTATCGTGGAAGG‐3′ (forward) and 5‘‐GCCATCACGCCACAGTTTC‐3′ (reverse). Relative expression of the target gene was calculated after normalization to that of the *GAPDH* housekeeping gene.

### 2.9 Enzyme‐linked immunosorbent assay

Conditioned medium collected from BPH‐1 cells cultured alone or with Molt‐3 cells in the absence or presence of tadalafil or KT5823 for 4 days was used to detect CCL5 secretion by the human CCL5 Quantikine ELISA Kit (DRN00B; R&D Systems) according to the manufacturer's protocol.

### 2.10 Statistical analysis

The data are expressed as the mean ± SD of at least three independent experiments. Differences between groups were analyzed by a paired *t* test. IHC data were analyzed by linear regression correlation or analysis of variance (ANOVA). *P* < 0.05 were considered as statistically significant. All statistical analyses were performed using SPSS 17.0 (SPSS Inc, Chicago, IL).

## 3 RESULTS

### 3.1 Infiltration of CD8^+^ T cells and expression of CCL5, CCND1, and PDE5 in human BPH prostate samples

**Figure 1:**
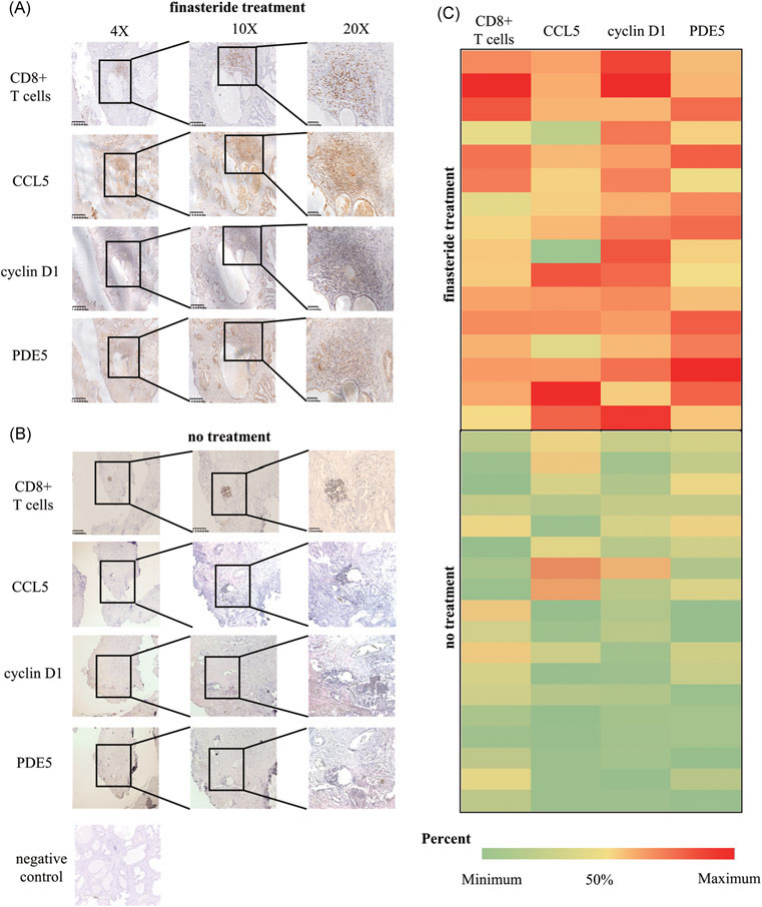
Comparison of CD8^+^ T‐cell infiltration and CCL5, CCND1, and PDE5 expression in tissues of finasteride‐treated and untreated BPH patients. A,B, Infiltration of CD8^+^ T cells and expression of CCL5, CCND1, and PDE5 were analyzed by IHC staining of serial paraffin sections from prostate tissues of BPH patients treated or not with finasteride for at least 6 months. C, Heat map showing the distribution of CD8^+^ T cells and CCL5, CCND1, and PDE5 expression in the finasteride‐treated and untreated groups. BPH, benign prostatic hyperplasia; IHC, immunohistochemistry; PDE5, phosphodiesterase type 5 [Color figure can be viewed at wileyonlinelibrary.com]

Our previous studies have shown that induction of CCL5 secretion by infiltrating CD8^+^ T cells promoted BEC proliferation in low androgen conditions.^10^ In the present study, we examined the infiltration of CD8^+^ T cells and expression of CCL5, CCND1, and PDE5 in BPH tissues from 16 patients treated or 18 patients not with finasteride for at least 6 months. The results indicated that the expression of CCL5, CCND1, and PDE5 was higher in tissues from finasteride‐treated patients than in those from untreated patients (CD8^+^ T‐cell infiltration, *P* < 0.0001; CCL5, *P* = 0.0002; CCND1, *P* < 0.0001; PDE5, *P* = 0.0001; one‐way ANOVA) (Figures 1 and S1A‐D). Linear regression correlation analysis revealed a positive correlation between CD8^+^ T‐cell infiltration and the expression of CCL5 (*r* ^2^ = 0.1444; *P* = 0.0266), CCND1 (*r* ^2 ^=^ ^0.4269; *P* < 0.0001), and PDE5 (*r* ^2^ = 0.3003; *P* = 0.0008) (Figure S1E‐G). So as between PDE5 and the expression of CCL5 (*r* ^2^ = 0.2605, *P* = 0.002), CCND1 (*r* ^2^ = 0.1969, *P* = 0.0086) (Figure S1H and S1I).

### 3.2 Activation of the cGMP/PKG pathway prevented the induction of BEC proliferation by CD8^+^ T cells

To investigate the influence of infiltrating CD8^+^ T cells on the growth of BECs in BPH samples from finasteride‐treated patients, we compared the infiltration of CD8^+^ T cells and the expression of CCND1, an indicator of cell growth, by IHC. The results showed that CD8^+^ T cells surrounded the epithelium area, whereas CCND1 was mainly expressed in BECs; furthermore, PDE5 expression was higher in the area of CD8^+^ T‐cell infiltration (Figure 1A and 1B).

**Figure 2:**
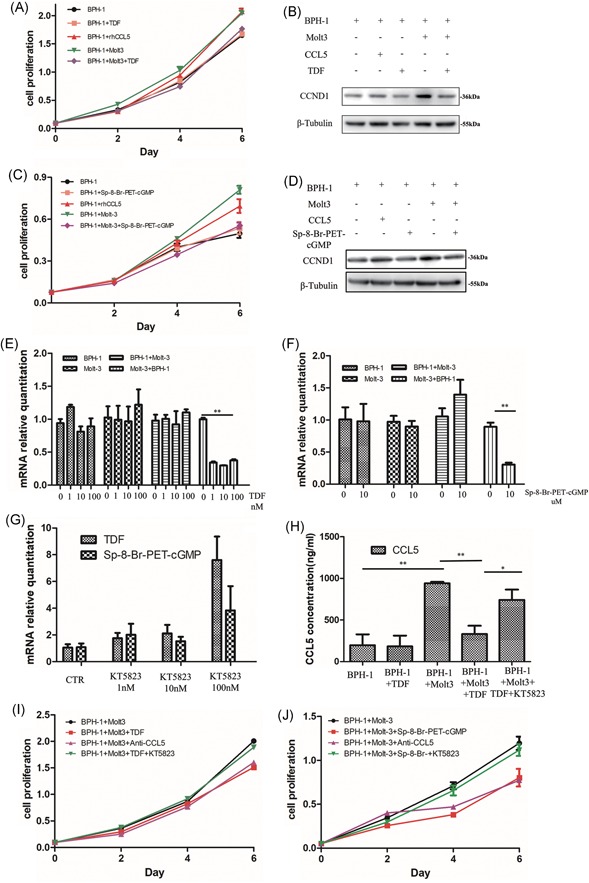
Activation of the cGMP/PKG signaling pathway could reverse the induction of BEC proliferation by CD8^+^ T cells through suppressing the secretion of CCL5 by CD8^+^ T cells in low androgen conditions. BECs were cocultured with or without Molt‐3 cells in low androgen conditions for 4 days. A,C, BECs cultured with rhCCL5 (1 μg/mL) and tadalafil (100 nM) or Sp‐8‐Br‐PET‐cGMP (10 μM) were analyzed for proliferation at days 2, 4, and 6 using the CCK‐8 assay. The data are shown as the mean ± SD; *P < 0.05 and **P < 0.01. B,D, BECs were harvested at day 4 and analyzed for CCND1 expression by Western blot analysis; β‐Tubulin was used as a loading control. E‐G, CCL5 mRNA expression in Molt‐3 cells at day 2 was analyzed by qPCR. CCL5 mRNA levels were downregulated by tadalafil (100 nM) or Sp‐8‐Br‐PET‐cGMP (10 μM) in Molt‐3 cells cocultured with BECs, but KT5823 (100 nM) reversed the effect. H, CCL5 secretion to conditioned medium of cocultures and monocultures treated or not with tadalafil and/or KT5823 at day 4 was assessed by ELISA. I,J, BPH‐1 cells treated or not with tadalafil or Sp‐8‐Br‐PET‐cGMP, anti‐CCL5 neutralizing antibody (2 μg/mL), and KT5823 were analyzed for proliferation at days 2, 4, and 6 using the CCK‐8 assay. Data are shown as the mean ± SD; *P < 0.05 and **P < 0.01. BEC, BPH epithelial cell; BPH, benign prostatic hyperplasia; CCK‐8, cell counting kit‐8; cGMP, cyclic guanosine monophosphate; ELISA, enzyme‐linked immunosorbent assay; mRNA, messenger RNA; PKG, protein kinase G; qPCR, quantitative polymerase chain reaction, TDF, tadalafil [Color figure can be viewed at wileyonlinelibrary.com]

We also examined the growth of BECs in vitro during coculture with CD8^+^ T lymphocytes (Molt‐3 cell line)^17^ using the CCK‐8 assay and Western blot analysis (Figure 2). The data revealed that Molt‐3 cells, as well as rhCCL5 (1 μg/mL), significantly promoted BEC proliferation in low androgen conditions at day 6 compared to monocultures (both *P* < 0.001, one‐way ANOVA; Figures 2A and S2A), so as the expression of CCND1 (Figure 2B). However, a PDE5‐I tadalafil or a PKG agonist Sp‐8‐Br‐PET‐cGMP decreased the proliferation of BECs cocultured with Molt‐3 cells compared to BEC monocultures or Molt‐3 cell monocultures in low androgen conditions (tadalafil, *P* = 0.003; Sp‐8‐Br‐PET‐cGMP, *P* = 0.001; one‐way ANOVA) (Figures 2A and 2C and S2A and S2B), so as the expression of CCND1 (Figure 2D). These results suggest that activation of cGMP/PKG signaling inhibits the proliferation of BECs in low androgen conditions through CD8^+^ T cells, but does not affect BECs directly.

### 3.3 Activation of cGMP/PKG signaling suppressed CCL5 secretion by CD8^+^ T cells in low androgen conditions

Our previous study has shown that CCL5 was the key chemokine secreted by CD8^+^ T cells to accelerate the proliferation of BECs^10^ and our present data indicated that CCL5 was involved in the crosstalk between BECs and Molt‐3 cells in coculture. Therefore, we analyzed the effect of tadalafil (100 nM) and Sp‐8‐Br‐PET‐cGMP (10 μM) on the expression of CCL5 mRNA in Molt‐3 cells by qRT‐PCR and found that the drugs significantly decreased CCL5 mRNA levels in Molt‐3 cells cocultured with BECs in low androgen conditions (*P* = 0.001 for both drugs by *t* test; Figure 2E and 2F). Consistent with these results, the secretion of CCL5 into conditioned medium of BPH‐1/Molt‐3 coculture was decreased by tadalafil (Figure 2H). These results indicate that activation of the cGMP/PKG pathway could inhibit CCL5 secretion by CD8^+^ T cells, and as a result, suppress the proliferation of BECs in coculture. Furthermore, an anti‐CCL5 neutralizing antibody (2 μg/mL) added to BEC/Molt‐3 cocultures prevented the induction of BEC proliferation (Figure 2I and 2J), which is consistent with the effect of rhCCL5 on BEC growth. However, a PKG inhibitor KT5823 could reverse the effect of tadalafil or Sp‐8‐Br‐PET‐cGMP on CCL5 expression (Figure 2G) and then suppress the proliferation of BECs in coculture (Figures 2I and 2J and S2C and S2D).

Thus, the activation of cGMP/PKG signaling could downregulate the secretion of CCL5 by CD8^+^ T cells, resulting in the inhibition of BEC proliferation.

### 3.4 cGMP/PKG activation downregulated NF‐κB phosphorylation in CD8^+^ T cells and CCL5/STAT5/CCND1 signaling in BECs

**Figure 3:**
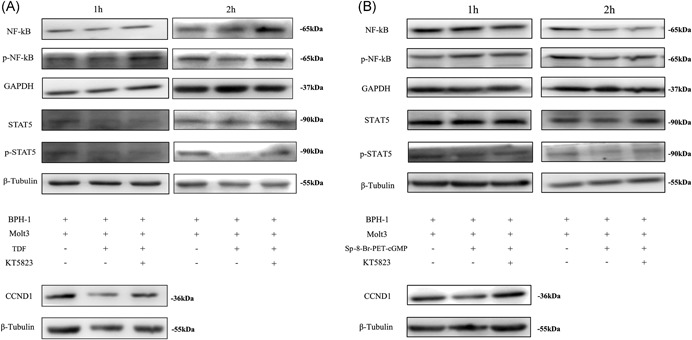
Expression of signaling molecules downstream of the cGMP/PKG pathway involved in the inhibition of BEC proliferation in low androgen conditions. Cocultures of BECs and Molt‐3 cells were treated with tadalafil (A) or Sp‐8‐Br‐PET‐cGMP (B) with or without KT5823 for 1 and 2 hours. BECs and Molt‐3 cells were harvested separately and analyzed by Western blot analysis for total NF‐κB and phospho‐NF‐κB (Molt‐3 cells) and total STAT5, phospho‐STAT5, and CCND1 (BPH‐1 cells). β‐Tubulin and GAPDH were used as loading controls. BEC, BPH epithelial cell; BPH, benign prostatic hyperplasia; cGMP, cyclic guanosine monophosphate; GAPDH, glyceraldehyde 3‐phosphate dehydrogenase; NF‐κB, nuclear factor‐κB; PKG, protein kinase G; STAT5, signal transducer and activator of transcription 5; TDF, tadalafil

Previous studies indicate that PDE5 inhibition by PDE5‐Is activates the cGMP/PKG signaling pathway in BECs.^18^, ^19^, ^20^ Therefore, we investigated whether the downregulation of CCL5 secretion by tadalafil and Sp‐8‐Br‐PET‐cGMP led to inhibition of signal transducer and activator of transcription 5 (STAT5) phosphorylation and CCND1 expression. Western blot analysis indicated that tadalafil (100 nM) or Sp‐8‐Br‐PET‐cGMP (10 μM) prevented the upregulation of STAT5 phosphorylation and CCND1 expression in BECs cocultured with Molt‐3 cells (Figure 3). However, KT5823 could reverse the effect of tadalafil or Sp‐8‐Br‐PET‐cGMP on STAT5 phosphorylation and CCND1 expression.

NF‐κB is a transcription factor playing a key role in inflammation, and several studies have demonstrated that it is regulated by the NO/cGMP/PKG signaling pathway.^21^, ^22^, ^23^ Therefore, we analyzed the phosphorylation of NF‐κB p65 subunit in the nuclear fraction of Molt‐3 cells treated with tadalafil/Sp‐8‐Br‐PET‐cGMP by Western blot analysis, which revealed that phospho‐NF‐κB p65 was downregulated by the drugs, but PKG inhibitor KT5823 (100 nM) reversed the effect (Figure 3).

Cumulatively, these results indicate that CD8^+^ T cells promoted BEC proliferation in low androgen medium through activation of the CCL5/STAT5/CCND1 signaling pathway.

### 3.5 Activation of cGMP/PKG signaling suppressed the secretion of CCL5 by CD8^+^ T cells and BEC proliferation in vivo

**Figure 4:**
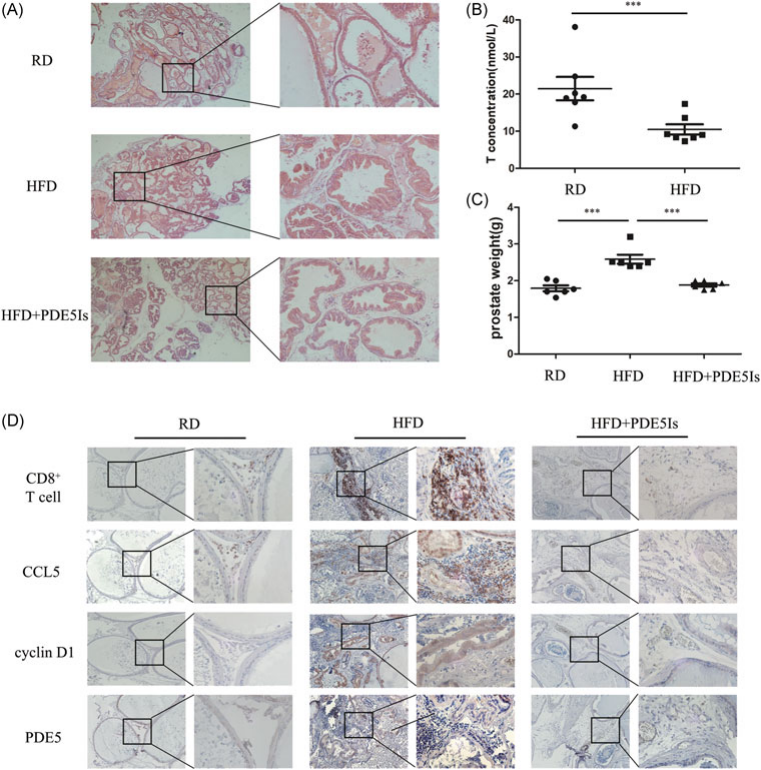
Activation of cGMP/PKG signaling suppressed CCL5 secretion by CD8^+^ T cells and reversed the induction of BEC proliferation in vivo. A, The degree of prostatic hyperplasia in rats receiving regular diet (RD), HFD, or HFD + PDE5‐Is was evaluated by hematoxylin and eosin staining of rat prostate samples (*n* = 6 rats per group). B, The serum testosterone levels of RD and HFD groups were measured using an automated chemiluminescence system at week 12; *P < 0.05, **P < 0.01, and ***P < 0.001 (by *t* test). C, All rat prostate weight of three groups were tested at last; *P < 0.05, **P < 0.01, and ***P < 0.001 (by one‐way ANOVA). D, CD8^+^ T‐cell infiltration and CCL5, CCND1, and PDE5 expression was analyzed by IHC staining in serial paraffin sections of the rat prostate. ANOVA, analysis of variance; BEC, BPH epithelial cell; cGMP, cyclic guanosine monophosphate; HFD, high‐fat diet; IHC, immunohistochemistry; PDE5, phosphodiesterase type 5; PKG, protein kinase G; RD, regular diet [Color figure can be viewed at wileyonlinelibrary.com]

To confirm the in vitro results, we investigated the effect of cGMP/PKG activation in CD8^+^ T cells in vivo using an HFD‐induced prostatic hyperplasia rat model. Analysis of prostate samples in three groups of rats (control, HFD, and HFD + PDE5‐Is) by hematoxylin and eosin staining revealed that HFD induced hyperplasia and low androgen conditions in the prostate (Figure 4A and 4B), indicating the successful establishment of the BPH model. The criteria of prostatic hyperplasia contained the layer number of epithelial cells and the protruding crest measured by hematoxylin and eosin staining, also including the weight of rat prostates (*P* < 0.001 for groups by *t* test, control vs HFD, and HFD vs HFD + PDE5‐Is; Figure 4C). The results of IHC analysis regarding the infiltration of CD8^+^ T cell and expression of CCL5, CCND1, and PDE5 in the rat prostate were consistent with the findings in vitro (Figure 4D).

## 4 DISCUSSION

A number of clinical trials have shown that PDE5‐Is used for the treatment of pulmonary arterial hypertension and erectile dysfunction^24^, ^25^, ^26^, ^27^ could effectively ameliorate LUTS associated with BPH.^3^, ^4^, ^5^ The main effect of cGMP/PKG signaling pathway associated with PDE5‐Is is considered to relax smooth muscle in the prostate.^6^ Moreover, Vignozzi et al^11^, ^14^ found that PDE5‐Is could inhibit inflammation levels in overall prostate, and we also considered that activation of the cGMP/PKG signaling pathway plays a role in inflammation. However, the specific signaling network underlying the regulatory role of the cGMP/PKG pathway in the inflammatory response in the prostate has not been clarified. Therefore, to further understanding of complex pathological mechanisms behind BPH development, in this study, we investigated the molecular cascade downstream of cGMP/PKG involved in the interaction between immune cells and BECs.

Prostate inflammation has been suggested as an etiological factor for BPH, and emerging evidence indicates that inflammation may contribute to prostate growth.^28^, ^29^ It was shown that downregulation of androgen receptor signaling in mouse prostate luminal cells could upregulate secretion of cytokines and chemokines in a cell‐autonomous manner and impair epithelial barrier function, leading to increased infiltration of immune cells into the prostate.^30^ Our previous studies have shown that in tissues of BPH patients treated with finasteride to lower androgen levels, CD8^+^ T‐cell infiltration was increased,^9^ which could accelerate the proliferation of BECs.^10^ Moreover, Szczypka et al^31^ found that the cGMP/PKG signaling pathway may control the activity of immune cells in the thymus, spleen, and mesenteric lymph nodes by increasing intracellular levels of cyclic nucleotides and that it is involved in the regulation of tumor‐specific immunity and autoimmune encephalomyelitis progression.^32^, ^33^, ^34^ Results obtained in a BPH animal model and clinical BPH patients treated with PDE5‐Is showed that the infiltration of inflammatory cells (pan‐leukocytes) into the prostate was significantly reduced.^11^, ^14^ Given these data and our preliminary results in a rat model regarding the link among cGMP/PKG signaling, CD8^+^ T‐cell infiltration, CCL5, and CCND1 expression, we considered that the cGMP/PKG/p65 pathway could inhibit the proliferation of BECs in low androgen conditions via downregulation of CCL5 secretion by CD8^+^ T cells.

First, the activation of the cGMP/PKG signaling pathway suppressed the growth of BECs indirectly by reducing CCL5 secretion in CD8^+^ T cells. Our in vitro results confirmed the functional significance of cGMP/PKG signaling pathway in BECs and lymphocytes by showing that activation of cGMP/PKG signaling suppressed BEC proliferation in the presence of CD8^+^ T cells but not in BEC monocultures (Figure 2). Furthermore, cGMP/PKG activation blocked CCL5 secretion by CD8^+^ T cells but not by BECs. In addition, a PKG inhibitor restored BEC proliferation and the secretion of CCL5 induced by CD8^+^ T cells in low androgen conditions.

To further investigate the molecular mechanism underlying CCL5 secretion by CD8^+^ T cells in low androgen conditions, we focused on the signaling cascade downstream of cGMP/PKG. Several studies indicated that NF‐κB plays an important regulatory role in the immune response,^21^, ^22^, ^23^ and that cGMP/PKG activation inhibited NF‐κB in hyperthermia‐exposed MCF‐7 cells, resulting in apoptosis.^21^ In this study, we showed, for the first time, that the cGMP/PKG pathway regulates the function of CD8^+^ T cells by controlling NF‐κB p65 phosphorylation, which affects the secretion of CCL5. A PKG inhibitor could re‐establish NF‐κB phosphorylation in CD8^+^ T cells and STAT5 phosphorylation and CCND1 expression in BECs, and restore BEC proliferation enhanced by CD8^+^ T cells, thereby implicating cGMP/PKG signaling in BPH pathogenesis.

Our previous studies deemed that there were more CD8^+^ T‐cell infiltrations in the finasteride treatment BPH tissues, and infiltrating CD8^+^ T cells accelerated the proliferation of prostate epithelial cells. Several clinical trials have reported that tadalafil and finasteride in combination for the treatment of LUTS resulted in significantly more patients achieving early clinical meaningful improvements in symptoms, and in greater treatment satisfaction versus placebo/finasteride.^35^, ^36^ Our results have shown that activation of cGMP/PKG signaling pathway associated with PDE5‐Is not only regulated the tone of the smooth muscle fibers of the prostate, urethra, and bladder, it may also downregulate inflammatory processes in LUT and exert antiproliferative effects in low androgen conditions.

However, some limitations of this study should be recognized. The major one is the deficiency of clinical BPH samples which were treated with both 5AR‐Is and PDE5‐Is. In addition, CD8^+^ T cells in rat prostate tissues were not separated by fluorescence‐activated cell sorting to further certify the function of the cGMP/PKG pathway.

## 5 CONCLUSIONS

**Figure 5:**
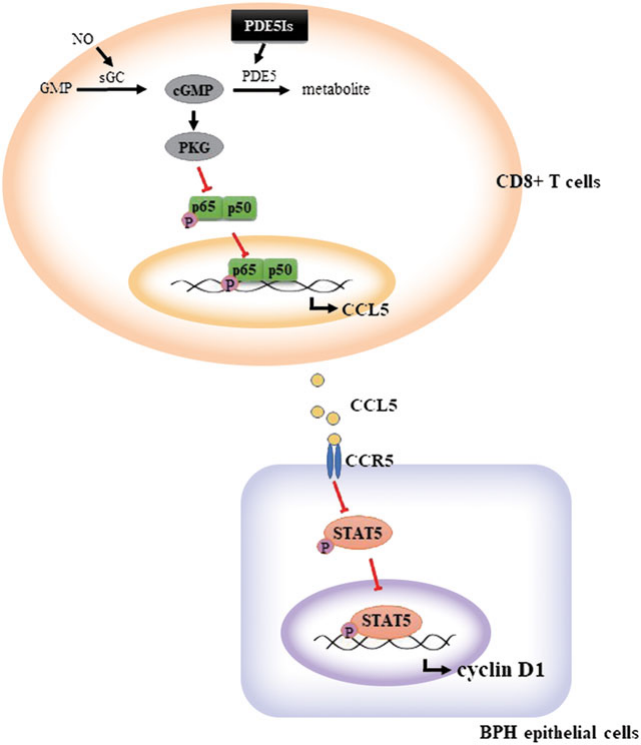
Schematic representation of the molecular mechanism underlying BEC proliferation in BPH. Activation of the cGMP/PKG signaling pathway in CD8^+^ T cells decreased NF‐κB phosphorylation and CCL5 secretion, resulting in the inhibition of CCL5/STAT5/CCND1 signaling in BECs and decrease of their proliferation in low androgen conditions. BEC, BPH epithelial cell; BPH, benign prostatic hyperplasia; cGMP, cyclic guanosine monophosphate; NF‐κB, nuclear factor‐κB; PDE5, phosphodiesterase type 5; PDE5‐I, PDE5 inhibitors; PKG, protein kinase G; STAT5, signal transducer and activator of transcription 5 [Color figure can be viewed at wileyonlinelibrary.com]

The important finding of the present study is that the activation of the cGMP/PKG/p65 pathway associated with PDE5‐Is in CD8^+^ T cells under low androgen conditions reduces the secretion of CCL5, which results in the inhibition of BEC proliferation via CCL5/STAT5/CCND1 signaling (Figure 5). As PDE‐Is block CCL5 secretion by CD8^+^ T cells and 5AR‐Is the reduce androgen levels, the combination of these drugs may effectively suppress the growth of BECs in the prostate, and therefore, can be considered as a candidate therapeutic approach for BPH patients in the future.

## CONFLICT OF INTERESTS

The authors declare that there are no conflict of interests.

## Supporting information

Supporting information

Supporting information

Supporting information
