# Supplementary figures and images for: Activation of cGMP/PKG/p65 signaling associated with PDE5‐Is downregulates CCL5 secretion by CD8 + T cells in benign prostatic hyperplasia
Source: Prostate. 2019 Apr 8;79(8):909–19. doi: 10.1002/pros.23801 (PMC6593656; doi:10.1002/pros.23801)

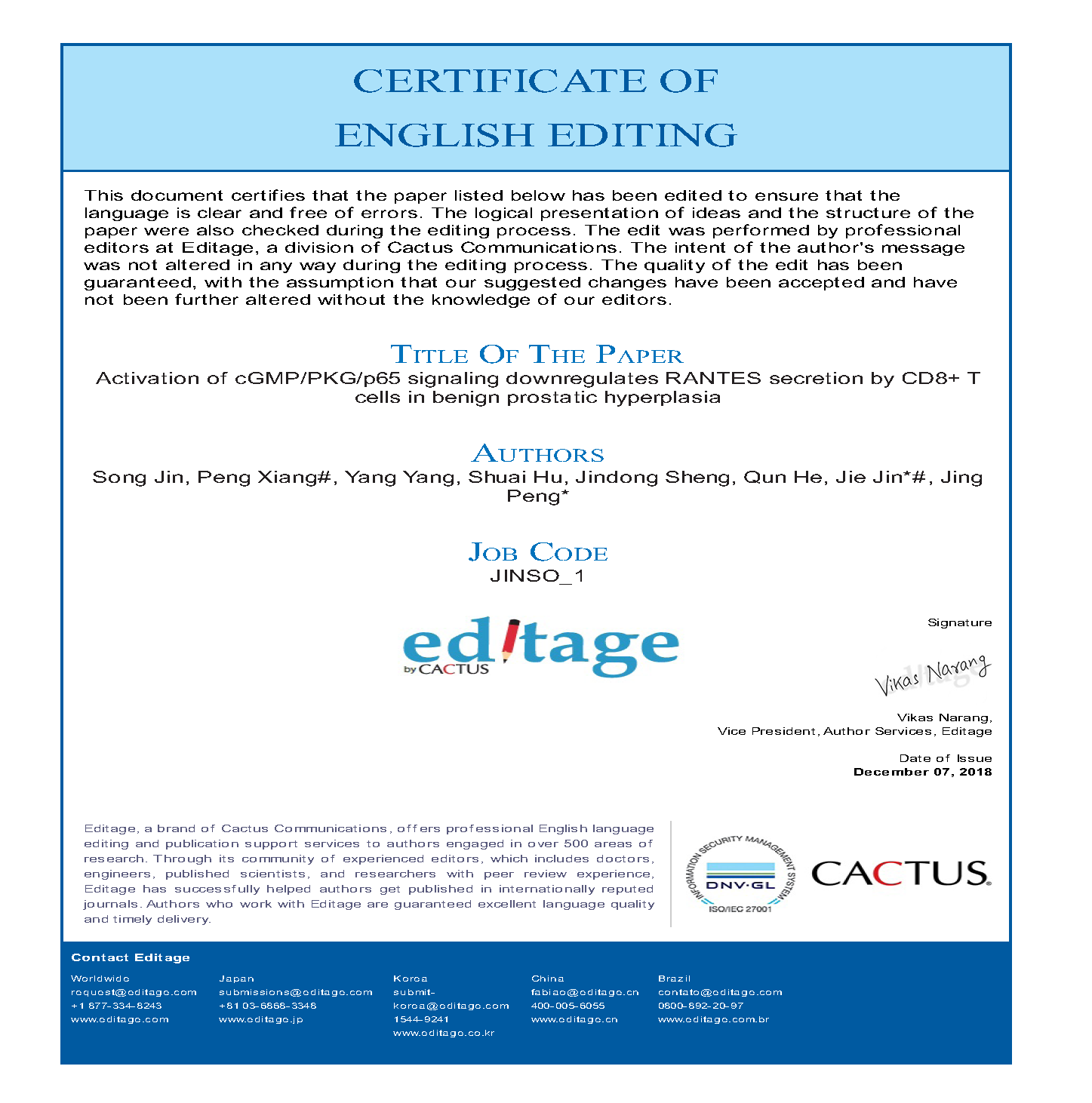

Supplement: Supplementary file 3 — Supporting information [file PROS-79-909-s003.tif]
